# Forkhead Box P3 Methylation and Expression in Men with Obstructive Sleep Apnea

**DOI:** 10.3390/ijms21062233

**Published:** 2020-03-23

**Authors:** David Sanz-Rubio, Arianne Sanz, Luis Varona, Rosa Bolea, Marta Forner, Ana V. Gil, Pablo Cubero, Marta Marin-Oto, Inmaculada Martin-Burriel, Jose M. Marin

**Affiliations:** 1 Translational Research Unit, Hospital Universitario Miguel Servet, Instituto de Investigación Sanitaria de Aragón (IISAragón), 50009 Zaragoza, Spain; davidsanzrubio91@gmail.com (D.S.-R.); mfornervicente@gmail.com (M.F.); victoriagilg@gmail.com (A.V.G.); jpcuberomarin@gmail.com (P.C.); marta.marin.oto@gmail.com (M.M.-O.); 2 Biochemical Genetics Laboratory (LAGENBIO), Instituto Agroalimentario de Aragón (IA2), Instituto de Investigación Sanitaria 0de Aragón (IISAragón), University of Zaragoza, 50013 Zaragoza, Spain; arianne@unizar.es; 3 Departamento de Anatomía Embriología y Genética Animal, Instituto Agroalimentario de Aragón (IA2), Universidad de Zaragoza, 50013 Zaragoza, Spain; lvarona@unizar.es; 4 Departamento de Patología Animal, Facultad de Veterinaria, Instituto Agroalimentario de Aragón (IA2), Universidad de Zaragoza, 50013 Zaragoza, Spain; rbolea@unizar.es; 5 Centro de Investigación Biomédica en Red de Enfermedades Respiratorias (CIBERes), 28000 Madrid, Spain; 6 Department of Respiratory Medicine, Clínica Universidad de Navarra, 31008 Pamplona, Spain; 7 Respiratory Service, Hospital Universitario Miguel Servet, University of Zaragoza, 50009 Zaragoza, Spain

**Keywords:** obstructive sleep apnea, FOXP3 methylation, FOXP3 expression, epigenetics.

## Abstract

Background: Epigenetic changes in obstructive sleep apnea (OSA) have been proposed as a mechanism for end-organ vulnerability. In children with OSA, Forkhead Box P3 (FOXP3) DNA methylation were associated with inflammatory biomarkers; however, the methylation pattern and its effect in the expression of this gene have not been tested in adults with OSA. Methods: Plasma samples from subjects without comorbid conditions other than OSA were analyzed (the Epigenetics Status and Subclinical Atherosclerosis in Obstructive Sleep Apnea (EPIOSA) Study: NCT02131610). In 16 patients with severe OSA (Apnea-Hypopnea Index—AHI- > 30 events/h) and seven matched controls (AHI < 5), methylation of FOXP3 gen was evaluated by PCR of the promoter and by pyrosequencing of the intron 1 Treg-specific demethylated region (TSDR). In another 74 patients with OSA (AHI > 10) and 31 controls, we quantified FOXP3 protein expression by ELISA and gene expression by quantitative real-time PCR. C-reactive protein (CRP) and plasma Treg cells were also evaluated. Results: Neither the levels of the promoter nor the TSDR demethylated region were different between controls and patients with OSA, whether they were grouped by normal or high CRP. FOXP3 protein and mRNA expression did not differ between groups. Conclusions: FOXP3 methylation or its expression is not altered in adults with OSA, whatever their inflammatory status.

## 1. Introduction

Obstructive sleep apnea (OSA) is a very prevalent sleep disorder characterized by recurrent episodes of partial or complete pharyngeal obstruction [1]. The repetitive collapse of the upper airway during sleep in OSA patients leads to recurrent arousals, intermittent hypoxia, and surges in sympathetic activity. These intermediate mechanics may explain, to some extent, the increased risk of cardiovascular morbidity and mortality that we and others have reported in patients with severe OSA [2,3]. In addition, oxidative stress and systemic inflammation are elevated in some OSA patients and diminish with effective therapy [4]. A cause-effect relationship between a systemic inflammatory state and cardiovascular disease has been suggested. In fact, interleukin-6 (IL-6) and C-reactive protein (CRP) are key inflammatory biomarkers associated with an increased risk of atherosclerosis and cardiovascular disease [5]. Systemic inflammatory variability responses in patients with OSA could be explained by different patterns of epigenetic modifications induced by the apneic episodes and, consequently, by the altered expression of genes involved in the atherosclerotic process. Hypoxia, a prominent characteristic of OSA, can induce hypermethylation of genes involved in cardiovascular diseases [6,7]. One of these genes, Forkhead Box P3 (FOXP3), controls the differentiation of lymphocytes into regulatory T lymphocytes (Treg), a subset of T helper cells that inhibit atherosclerosis by modulating lipoprotein metabolism [8]. Increased methylation in the promoter region of the *FOXP3* gene has been described in children with OSA and systemic inflammation [9]. In this study, *FOXP3* DNA methylation levels were closely correlated with CRP levels, indicating a potential mechanistic link. On the other hand, *FOXP3* mRNA expression has been found to be decreased in Chinese adults with OSA compared to non-OSA controls [10]. However, only patients with OSA and severe daytime hypersomnia were included in the latter study, and no methylation levels of the *FOXP3* gene were measured. In a recent whole-genome DNA methylation analysis of patients with and without OSA, Chen YC et al. found a number of differences in methylation levels in several genes between cases and controls in the discovery sample, but in the validation cohort, no significant difference in DNA methylation levels of those selected genes was found [11]. No study has simultaneously evaluated subclinical atherosclerosis with epigenetics and immunological changes in patients with OSA.

The Epigenetics Status and Subclinical Atherosclerosis in Obstructive Sleep Apnea (EPIOSA) Study is a longitudinal study with the main objective of identifying epigenetic markers associated to the prevalence and progression of subclinical atherosclerosis in individuals with OSA without co-morbid conditions (ClinicalTrials.gov: NCT02131610) [12]. We hypothesized that adults with OSA may have different systemic inflflammatory responses reflflecting different patterns in *FOXP3* gene methylation and expression.

## 2. Results

### 2.1. Subject Characteristics

A brief flowchart of the subjects’ selection is shown in Figure S1. Among 1291 consecutive subjects who came for evaluation to the Sleep Unit for suspected OSA during the period from 2015 to 2016, 862 did not met all inclusion/exclusion criteria, and 55 refused to participate. For the purpose of this study, we also excluded women, so 128 participants were included in this study. Baseline characteristics of consecutive patients included (*n* = 128) and not included in this study are shown in Supplementary Table S1. To assess differences in *FOXP3* methylation levels in OSA versus non-OSA subjects, we selected the first 16 patients with severe OSA (Apnea-Hypopnea Index—AHI > 30) and seven healthy subjects (AHI < 5) matched by age (± 2 years) and body mass index (BMI) (± 2 kg/m^2^). In the rest of the cohort (31 controls and 74 with OSA), the FOXP3 protein and gene expression were evaluated.

### 2.2. FOXP3 DNA Methylation Analysis

The methylation level of two DNA fragments of the *FOXP3* gene was analyzed in seven controls and 16 OSA patients divided into two groups based on their levels of high-sensitive C-reactive protein (hsCRP). The characteristics for those subjects are shown in Table 1. Patients with OSA had a very severe disease according with AHI, with no difference between those with low and high-hsCRP (65.1 ± 23.7 vs. 69.2 ± 17.6 events/h, *p* > 0.05). They also had a higher intima-media thickness (IMT) compared to controls regardless of hsCRP plasma levels. Otherwise, no significant differences existed between groups in age, BMI, and plasma lymphocyte Tregs.

**Figure 1:**
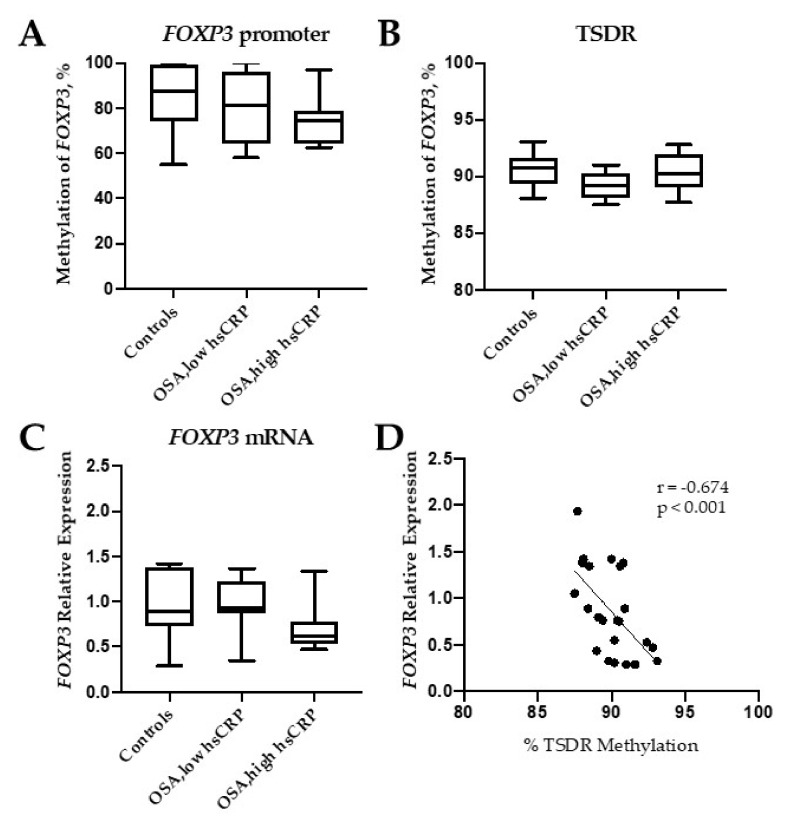
Methylation of Forkhead Box P3 (*FOXP3)*. Percentage of methylated DNA in the promoter (**A**), in the Treg-specific demethylated region (TSDR) (**B**), and FOXP3 mRNA expression levels (**C**) in controls (healthy subjects) and obstructive sleep apnea (OSA) patients with low and high high-sensitive C-reactive protein (hsCRP) blood levels. Correlation between TSDR methylation and *FOXP3* mRNA expression (**D**). Box and whiskers represent median and 5-95 percentile values.

The methylation levels of the promotor (containing five CpG sites) were similar in controls and OSA patients (Figure 1A). We then performed pyrosequencing analysis within the intronic 1 region of the *FOXP3* gene, which contains the Treg-specific DNA demethylation region (TSDR), and found that the mean methylation was not modified in OSA patients (Figure 1B). The methylation levels of the 11 individual CpG sites did not differ between OSA and control subjects. In accordance to this absence of methylation changes, *FOXP3* mRNA expression did not change between groups (Figure 1C). Whereas no correlation was observed between *FOXP3* promoter methylation levels and the transcript expression of this gene, as expected, TSDR methylation showed a negative Pearson correlation with the transcript *FOXP3* mRNA expression in all 23 individuals (*r* = −0.674, *p* < 0.001) (Figure 1D). In a secondary analysis, using log *FOXP3* DNA methylation levels instead of the absolute values did not change the results.

### 2.3. FOXP3 Protein and Gene Expression

The FOXP3 protein was quantified in the plasma samples of the subjects in which their methylation was evaluated. No significant differences were observed between controls and OSA patients and neither between patients with low hsCRP and high hsCRP (Supplementary Figure S2A). In this set of patients, gene expression showed a positive Spearman correlation (*r* = 0.365; *p* = 0.031) with the protein expression (Supplementary Figure S2B).

In the second phase of the study, we develop a wider gene expression analysis with the rest of the participants: 31 healthy controls and 74 OSA patients grouped as mild-moderate OSA (AHI = 10 to 29) and severe OSA (AHI ≥ 30). Baseline characteristics of these groups are shown in Table 2. Patients with severe OSA showed a slightly higher BMI and higher arterial blood pressure. Additionally, the levels of ApoB and hsCRP were higher compared to healthy subjects. Age and Treg were similar across the three groups (*p* > 0.05).

**Figure 2:**
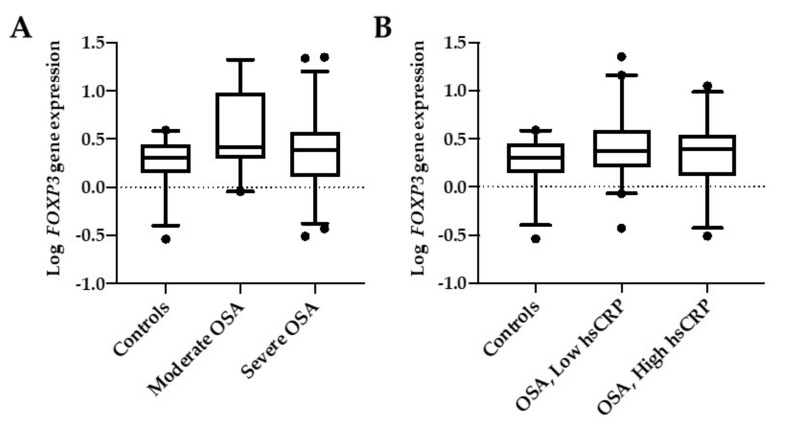
*FOXP3* mRNA expression. Comparison between controls and OSA groups according to their AHI (**A**) or their levels of hsCRP (**B**). Moderate OSA are patients with AHI <30, and severe OSA are patients with AHI ≥30. OSA low-hsCRP are patients with serum hsCRP < 0.3 mg/dl, and OSA high-hsCRP are patients with serum hsCRP > 0.3 mg/dl. Box and whiskers represent median and 5-95 percentile values.

*FOXP3* mRNA expression levels in this cohort were not significantly different between controls and the two OSA groups divided according to AHI (Figure 2A) or when they were grouped according with their hsCRP levels (Figure 2B).

### 2.4. Association of FOXP3 mRNA Expression with Clinical Data

**Figure 3:**
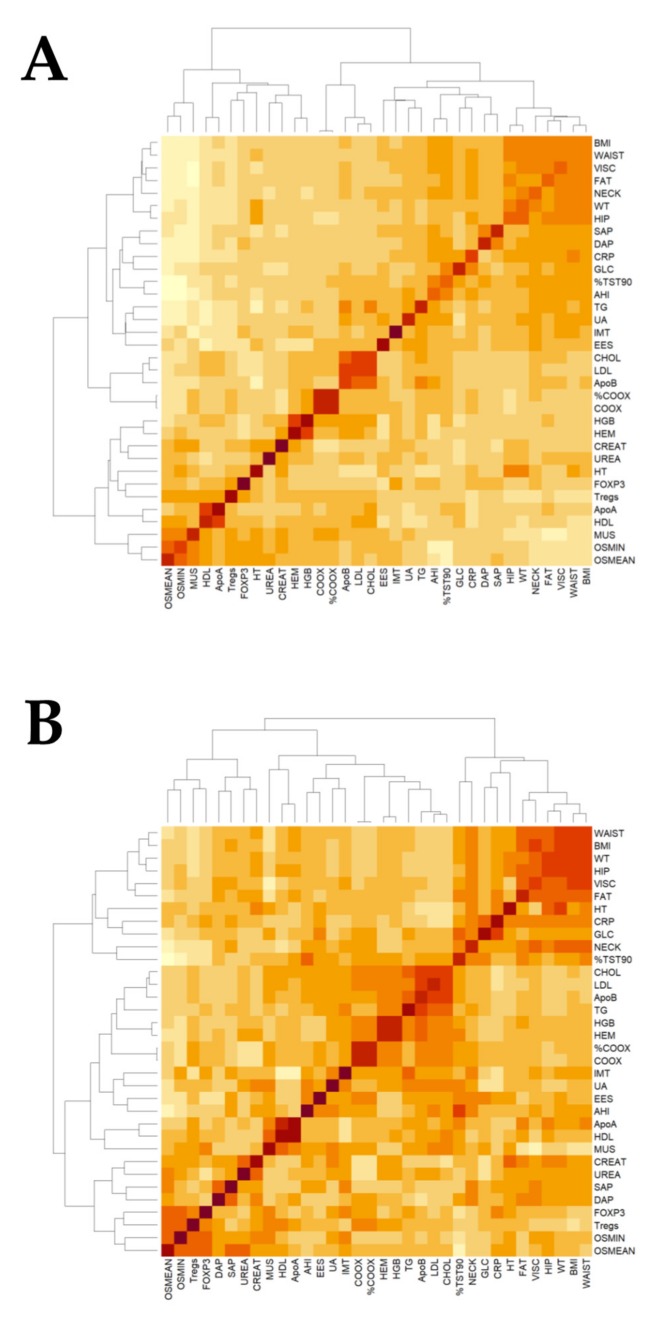
Heatmap of clinical variants. Plots showing the relationship between clinical variants in the Epigenetics Status and Subclinical Atherosclerosis in Obstructive Sleep Apnea (EPIOSA) cohort (**A**) and in OSA patients (**B**). BMI = body mass index, WT = weight, HT = height, FAT = body fat VISC = visceral fat, % TST90 = percentage of total sleep time with a percentage of O_2_ saturation lower than 90%, AHI = Apnea-Hypopnea Index, UA = uric acid, EES = Epworth sleepiness scale, CRP = high-sensitive C-reactive protein, GLC = glucose, SAP = systolic blood pressure, DAP = diastolic blood pressure, CHOL = cholesterol, LDL = low-density lipoprotein, Apo = apolipoprotein, HGB = hemoglobin, HEM = hematocrit, COOX = Co-oximetry, TG = triglycerides, IMT = intima-media thickness of the common carotid artery, CREAT = creatinine, HDL = high-density lipoprotein, MUS = muscle, Tregs = T regulatory lymphocytes, O_2_ MIN = lowest oxygen saturation, and O_2_ MEAN = mean oxygen saturation.

The heatmap of Figure 3 shows the correlations of clinical, biochemical, and epigenetic data for the cohort as a whole (Figure 3A) and for patients with OSA (Figure 3B), with the darkest red colors revealing a stronger correlation between parameters. In the multiple regression model, after controlling for age and BMI, *FOXP3* expression was positively related with Treg in the total cohort (*r* = 0.263; *p* = 0.023) and in the OSA group (*r* = 0.238; *p* = 0.033). However, % DNA methylation, neither the promotor nor the TSDR regions, and *FOXP3* expression were independently associated with variables reflecting OSA severity, such as AHI or CT90 %, in the whole cohort or among the OSA group.

As shown in Table 2, there was a trend towards higher IMT and a proportion of subjects with subclinical atherosclerosis in patients with OSA. Compared to healthy subjects, this difference reached statistical significance for severe OSA patients. The heatmaps also shown a strong positive correlation between ApoB, LDL, and cholesterol in the total set of individuals and between triglycerides, AHI, and CT90 % in OSA patients. In the multiple regression analysis, when including the models’ ages, BMI, systolic, and diastolic blood pressures, none of these variables are independent predictors of IMT or subclinical atherosclerosis in the whole cohort or the OSA group.

## 3. Discussion

As far as we know, this is the first study conducted in adults with OSA to assess the methylation status of the *FOXP3* gene. In our initial study with 23 individuals, methylation of the gene promoter in adults with severe OSA did not diverge from controls, and differences were neither observed between OSA groups with distinct hsCRP values. Similarly, methylation of the TSDR region was not altered in patients with OSA. In accordance with this lack of regulation, the *FOXP3* mRNA and protein expression did not vary significantly between groups. On the other hand, our study reaffirms the association between OSA and the risk of accelerated atherosclerosis regardless of the presence or absence of systemic inflammation.

The promoter region of the *FOXP3* gene is demethylated in both naïve and memory thymic-derived Treg cells [13]. Epigenetic modifications, such as the transcriptional silencing of *FOXP3*, via hypermethylation of CpG islands in the promoter and intronic regions, have been associated with poor outcomes in children with asthma. In this sense, it has been reported as an increase in asthma severity in parallel with *FOXP3* hypermethylation in blood DNA and diminished Treg function in children exposed to diesel exhaust particles [14,15]. In adults with systemic sclerosis [16], and a peanut allergy [17], *FOXP3* hypermethylation seems to play also an important mechanistic role. OSA is becoming a pandemic in parallel with the increasing prevalence of obesity. Given the great phenotypic variability in the clinical presentation of patients with OSA and in their prognosis, it would be desirable to have robust health-predicting biomarkers. Evaluating the epigenetic modifications and, specifically, DNA methylation patterns is a promising area of research that can offer novel diagnostic biomarkers and targeted therapeutics tools to identifying and personalizing the treatment for the most vulnerable individuals. Hypermethylation of the *FOXP3* gene occurs in children suffering OSA and systemic inflammation [9], and it was suggested that this epigenetic modification could lead to a downregulation of *FOXP3* and the subsequent reduction of the number of Treg cells. In fact, this type of cell is significantly reduced in children with moderate-severe OSA [9,18]. In accordance, in a study developed in Chinese adult OSA patients, the expression of *FOXP3* was shown to be downregulated in OSA patients, with this decrease higher in severe OSA, those patients also displaying a decrease of circulating Tregs [10]. However, in that study, the methylation profile of this gene was not investigated, and the blood levels of Treg/CD4+ were particularly low (controls: 2.81 ± 0.46 and OSA: 1.50 ± 0.38). In any case, the interpretation of results in studies of association between OSA and health consequences should consider the phenotypic differences of OSA in the Chinese population with respect to the Caucasian population [19].

Male gender, together with obesity, aging, upper airway anatomy, smoking, alcohol, and genetic predisposition, are the main risk factors that increase vulnerability to OSA [20]. We present here a study of the *FOXP3* gene in a Spanish OSA cohort, which includes methylation, protein, and gene expression profiles and their relationship with apnea parameters, immunological, and subclinical atherosclerosis data. This cohort were carefully selected to exclude subjects with any comorbid condition, and for the purpose of this analysis, we only included men to avoid any sex influence [21]. We have analyzed methylation in two different regulatory regions of the *FOXP3* gene, located in the promoter and in the Treg-specific demethylated region, and contrary to what happens in children with OSA, we have not found differences in *FOXP3* methylation or Treg levels between patients with OSA and healthy subjects, even when patients were grouped according with the coexistence of systemic inflammation. This discordance between children and adult OSA patients could be due to the relatively mild inflammation response observed in our patients, even in those classified in the group “high hsCRP” (mean hsCRP = 0.64 mg/dL), compared to children with “high hsCRP” (hsCRP > 1.5 mg/dL) [9]. Nevertheless, the hsCRP levels observed in our study are like those reported in adults with OSA in other studies, where individuals with severe OSA display hsCRP levels > 0.3mg/dL [22].

We could not confirm the findings of Tan et al., which demonstrated an increase in *FOXP3* methylation [10]. This could have been due to the limited number of individuals used in the methylation study. As methylation of the TSDR region was negatively correlated with *FOXP3* mRNA expression and gene and protein expression correlated positively, we quantified the expression of this gene in blood from a larger set of subjects (*n* = 105). Besides the trend to Treg reduction observed in the group of patients with high hsCRP, *FOXP3* expression was not modified by the inflammatory condition in the EPIOSA group.

Our study presents certain limitations that prevent a generalization of our results to clinical practice. First, it does not include women. In this study, we tried to match adults with/without OSA as strictly as possible, and different processes could increase the variability of FOXP3 expression in women. As it is shown in Table 1, the average age of the patients who come to our sleep clinic is 43 years old. This means that most women of this age are premenopausal or menopausal, a process that has been linked with changes in DNA methylation in different tissues, including blood [23,24]. Moreover, FOXP3 is located in the X chromosome, which suffers imprinting in females. Previous research has shown differences in FOXP3 methylation depending on the X copy inactivated, which could explain a lower expression of this gene in women and increase the variability of FOXP3 expression in this sex [25]. Therefore, in this first study, we included only men, and if there had been differences in the degree of methylation of the FOXP3 gene, then it was planned to evaluate these differences in women with/without OSA taking into account their age and hormonal status. Second, our study only includes patients with OSA without comorbidities or cardiovascular risk factors. The participants studied here with severe OSA are not entirely representative of adult patients who come to the sleep units. Patients with severe OSA (AHI > 30) were selected to enhance possible changes in the methylation profile. However, our patients characteristically showed snoring and severe nocturnal hypoxia. Hypoxia, especially its "intermittent" characteristic, has been invoked as one of the intermediate mechanisms that explain the morbidity and mortality in OSA. However, the molecular route by which this association is explained is far from being elucidated. Hypoxia at the molecular level stimulated hypoxia-inducible factor (HIF) transcription factors HIF-1α and HIF-2α [26]. Recent data in in vitro and in animal models indicate that *FOXP3* is a direct HIF-1α target gene; that hypoxia, through HIF-1α, overexpresses this gene and promotes Treg production and function in vitro and in vivo [27]. To date, there are no studies in humans that have definitively elucidated the role of local or general hypoxia and its relationship to atherosclerosis or the role that would play the activity (repressed or stimulated) of *FOXP3*. Another limitation of our study is that we have only evaluated the respiratory variables. Other fundamental events that occur during sleep in patients with OSA, such as microarousals or dysfunction of the autonomic nervous system activity, could determine epigenetic modifications.

A more general alternative explanation of our results with those found in children with OSA is that children and adults could have different epigenetic modifications compared to the same common internal aggression, such as the repetitive obstructive events of the upper airway that characterize OSA. Future studies should evaluate these relationships, and more studies are necessary to identify intermediate mechanisms that relate OSA to premature cardiovascular diseases and to develop new biomarkers that can help in the phenotyping of these patients.

## 4. Materials and Methods

### 4.1. Study Population

For the purpose of this study, we included consecutive participants recruited at the Sleep Clinic of the Hospital Universitario Miguel Servet during the first year of the ongoing EPIOSA study. We included subjects aged 20 to 60 years old and free of any acute or chronic comorbid condition other than OSA. Detailed inclusion and exclusion criteria are provided in Supplementary Table S1. The study was approved by the Regional Institutional Review Board of Aragon, Spain (IRB#03/2013, February 13, 2013), and all participants gave written informed consent before any procedure was done.

### 4.2. Clinical Assessment

Demographic, anthropometric, and clinical data were obtained at recruitment using specific questionnaires and standard measurements [12]. Daytime somnolence was assessed with the Epworth test [28]. All subjects underwent home sleep testing, as reported previously [12]. Trained personnel manually scored polygraph data in accordance with American Academy of Sleep Medicine guidelines [29]. The Apnea-Hypopnea Index (AHI) was calculated based on the average number of apnea plus hypopnea episodes per hour of recorded sleep time. The cut-off value of AHI for the diagnosis of OSA was 10 due to the possible underestimation caused by the use of home sleep studies [30]. Blood samples were taken in the morning after the sleep study. Within two hours after collection, hsCRP was measured using automated immunonephelometry (Behring Nephelometer II Analyzer, Dade Behring, Germany). The cut-off of the normal value of hsCRP was 0.3 mg/dL. Blood in EDTA and Paxgene W tubes were stored at −80 °C for DNA and RNA analysis. Common carotid intima-media thickness (CIMT) was assessed using the Philips IU22 ultrasound system (Philips Healthcare, Bothell, WA, USA). Ultrasound images were acquired with linear high-frequency 2-dimensional probes (Philips Transducer L9-3, Philips Healthcare), using the Bioimage Study protocol for the carotid arteries [31]. Examination of the carotid territory included the terminal portion (10 mm) of the common carotid, the bulb, and the initial portion (10 mm) of the internal and external carotid arteries. Subclinical atherosclerosis was defined in subjects with a CIMT greater than the upper limit (75 percentile) of the normal distribution of the maximum CIMT by segments of age groups as reported in our local healthy population [32] or by the presence of at least ≥1 plaque of atheroma.

### 4.3. Flow Cytometry

Peripheral blood mononuclear cells (PBMCs) were isolated from fresh, whole blood by density gradient centrifugation using Ficoll-Hypaque (Sigma-Aldrich, St Louis, MO, USA). CD4+ T cells and CD4+CD25+CD127dim/- Tregs were purified from PBMC using a CD4+ Cell Isolation Kit (Miltenyi, Bergisch Gladbach, Germany) and CD4+CD25+CD127dim/- Regulatory T Cell Isolation Kit II (Miltenyi) according to the manufacturer’s instruction, respectively.

### 4.4. FOXP3 DNA Methylation Analyses

DNA was isolated from frozen whole blood using an Illustra Blood GenomicPrep Midi Flow Kit (GE Healthcare, Little Chalfont, UK). The methylation status of *FOXP3* was analyzed in two different regions of this gene, the promotor and the Treg-specific DNA demethylated region (TSDR) in intron 1. DNA methylation of the FOXP3 promoter was determined using a high-resolution melt-polymerase chain reaction (HRM-PCR). Bisulfite-treated DNA was amplified using primers designed by MethPrimer [33] to spam fragments of the *FOXP3* promoter (position 49264826 to 49264661 at the Homo sapiens chromosome X, GRCh38.p2 Primary Assembly (ref. NC_000023.11), and at 165 bp from the transcription start, containing 5 potential CpG islands. Primers and specific methodology for this analysis is described in the Supplementary Materials. The methylation of the TSDR located in *FOXP3* intron 1 was also evaluated. The fragment analyzed includes 11 CpG sites, and total TSDR methylation was calculated as the mean of methylation percentages of each individual CpG. Methylation in every single CpG site was quantified by pyrosequencing using the assay ID ADS 783 (Ensembl Transcript ID: ENST00000376207) through EpigenDX (Worchester, MA, USA), a company specializing in genomic methylation assays.

### 4.5. FOXP3 Gene Expression

Total RNA was obtained from whole blood. Two-step TaqMan quantitative real-time PCR (qRT-PCR) was performed to determine the *FOXP3* expression in OSA patients and controls. Specific methodology for RNA extraction from whole blood, Taqman assays used in qRT-PCR, and data normalization are shown in the Supplementary Materials.

### 4.6. Quantification of FOXP3 Protein

The amount of FOXP3 was measured in plasma by sandwich enzyme-linked immune-sorbent assay technology using a FOXP3 ELISA Kit for humans (Aviva Systems Biology, San Diego, CA, USA) and following the manufacturer’s instructions. Fluorescence was measured in a Synergy HT reader (BioTek Instruments, Winooski, VT, USA). Standard curve was prepared using a FOXP3 control provided by the kit, and plasma signals were interpolated in it to get the FOXP3 quantification. The intra- and inter-assay precision coefficients of variation (CV) assessed by the kit were 5.7% and 9.5%, respectively.

### 4.7. Statistical Analysis

Continuous variables were summarized as means ± SD or medians (and interquartile ranges) and categorical variables as proportions. Comparisons between groups were done by means of an independent Student’s *t*-test if the data were normally distributed and a Mann–Whitney U test if not. The chi-square test was used to analyze categorical variables. Relative gene expression values were log-transformed for further analysis. Given its nongaussian nature, we used a log-linear model to identify relationships between *FOXP3* expression levels and clinical parameters, considering the effects of age. In the secondary analysis, log *FOXP3* DNA methylation levels, instead of the % methylation of the *FOXP3* gene, were used to evaluate differences between healthy controls and OSA patients. In order to illustrate the raw relationships between variables, we calculated the correlations between variables using the heatmap function in the R package. The association of the presence of plaques in the carotid arteries with the % of DNA methylation, log FOXP3 expression, or Treg lymphocytes was studied with logistic regression models and adjusted for age and BMI (as linear variables) and traditional risk factors. The free software R.2.15 was used for developing all analyses (www.r-project.org).

## 5. Conclusions

Our work confirms the tendency to premature development of subclinical atherosclerosis in patients with OSA without other associated cardiovascular risk factors. We also found that the *FOXP3* transcript expression in blood is associated with Treg content, but contrarily to the studies performed in children, we cannot associate this marker with other systemic inflammation parameters or with subclinical atherosclerosis. Then, *FOXP3* expression and/or methylation cannot be used as a biomarker of systemic inflammation or severity in adult OSA patients

## Figures and Tables

**Table 1:** Characteristics of healthy controls subjects and obstructive sleep apnea (OSA) patients (with low or high c-reactive proteins) in the Forkhead Box P3 (*FOXP3)* DNA methylation analysis.

Variable	Controls	OSA	OSA (low hsCRP)	OSA (high hsCRP)
Individuals, n	7	16	8	8
Age (years)	41.9 ± 6.9	43.2 ± 9.3	45.1 ± 9.6	42.4 ± 9.2
BMI (kg/m^2^)	29.8 ± 2.8	31.6 ± 4.0	30.7 ± 3.5	32.7 ± 4.4
SBP (mmHg)	123 ± 9	133 ± 15 *	132 ± 17	133 ± 19
DBP (mmHg)	69 ± 9	82 ± 11 *	81 ± 14	82 ± 13
AHI (events/hour)	2.2 ± 2.5 **	67.2 ± 20.3	65.1 ± 23.7	69.2 ± 17.6
Nadir SaO_2_	89.6 ± 2.4 **	73.2 ± 8.1	71.4 ± 7.7	75.1 ± 8.5
CT90, %	0.1 ± 0.3 **	45.2 ± 24.7	43.2 ± 25.1	43.3 ± 25.7
ESS	9.7 ± 6.1	12.6 ± 3.5	12.7 ± 3.9	12.6 ± 2.8
hsCRP (mg/dl)	0.08 ± 0.09 *	0.35 ± 0.25	0.14 ± 0.09	0.56 ± 0.17 ***
ApoA (mg/dl)	140.9 ± 21.1	141.4 ± 20.3	140.2 ± 25.3	144.3 ± 25.3
ApoB (mg/dl)	96.9 ± 24.4	114.1 ± 29.3	112.3 ± 29.9	117.6 ± 32.2
IMT (mm)	0.54 ± 0.09 *	0.66 ± 0.12	0.61 ± 0.09	0.70 ± 0.15
Tregs (% CD4+ cells)	6.7 ± 1.5	7.1 ± 1.5	6.5 ± 1.1	7.6 ± 1.7

AHI = apnea-hypopnea index, BMI = body mass index, hsCRP = high-sensitive C-reactive protein, nadir SaO_2_ = lowest oxygen saturation, CT90 = percentage of total sleep time with a percentage of O_2_ saturation lower than 90%, DBP = diastolic blood pressure; ESS = Epworth sleepiness scale, IMT = intima-media thickness of the common carotid artery, SBP = systolic blood pressure, Apo = apolipoproteine, and Tregs = T regulatory lymphocytes. All values are expressed as mean ± standard deviation. Differences between controls and OSA groups: * *p*-value < 0.05, and ** *p*-value < 0.001. Differences between OSA groups: *** *p*-value < 0.001.

**Table 2:** Characteristics of health controls subjects and patients with mild-moderate or severe OSA used in the gene expression study.

Variable	Controls	Mild-moderate OSA	Severe OSA
Individuals, n	31	19	55
Age (years)	41.5 ± 8.4	45.3 ± 6.8	44.1±8.9
BMI (kg/m^2^)	27.2 ± 3.1	30.9 ± 4.6	32.2 ±5.2 ***
AHI (events/hour)	2.0 ± 2.9	20.5 ± 6.5 *	57.7 ± 19.9 *** ^§^
ESS	10.3 ± 4.7	8.8 ± 4.5	11.1 ± 4.6
SBP (mmHg)	122 ± 10	128 ± 10	132 ± 15 **
DBP (mmHg)	73 ± 10	80 ± 8*	83 ± 11 **
hsCRP (mg/dl)	0.13 ± 0.12	0.26 ± 0.28	0.40 ± 0.34 *
ApoA (mg/dl)	142.9 ± 22.1	152.2 ± 25.4	143.3 ± 22.5
ApoB (mg/dl)	98.8 ± 23.9	105.6 ± 27.4	117.8 ± 27.8 *
TREG	7.1 ± 1.9	7.2 ± 1.5	7.1 ± 1.5
IMT (mm)	0.56 ± 0.09	0.61 ± 0.15	0.65 ± 0.13 *
Subclinical atherosclerosis, n (%)	5 (16.1)	4 (21.0)	20 (36.3) **^§^

All values are expressed as mean ± standard deviation. Differences between controls and OSA groups: * *p*-value < 0.05, ** *p*-value < 0.01, and *** *p*-value < 0.001. Differences between OSA groups: *p* < 0.05 and § *p* < 0.01.

## Supplementary Materials

Supplementary materials can be found at https://www.mdpi.com/1422-0067/21/6/2233/s1.

## Abbreviations

OSA	Obstructive sleep apnea
FOXP3	Forkhead Box P3
AHI	Apnea-Hypopnea Index
IMT	Intima-media thickness of the common carotid artery
